# The Microbiome and Alzheimer’s Disease: Potential and Limitations of Prebiotic, Synbiotic, and Probiotic Formulations

**DOI:** 10.3389/fbioe.2020.537847

**Published:** 2020-12-14

**Authors:** Karan Arora, Miranda Green, Satya Prakash

**Affiliations:** ^1^Biomedical Technology and Cell Therapy Research Laboratory, Department of Biomedical Engineering, Faculty of Medicine, McGill University, Montreal, QC, Canada; ^2^Department of Bioengineering, Faculty of Engineering, McGill University, Montreal, QC, Canada; ^3^Biena Inc., Saint-Hyacinthe, QC, Canada

**Keywords:** microbiome, probiotics, prebiotics, Alzheimer’s disease, gut-brain axis, neurological disorders, synbiotics

## Abstract

The Microbiome has generated significant attention for its impacts not only on gastrointestinal health, but also on signaling pathways of the enteric and central nervous system via the microbiome gut–brain axis. In light of this, microbiome modulation may be an effective therapeutic strategy for treating or mitigating many somatic and neural pathologies, including neurodegenerative disorders. Alzheimer’s disease (AD) is a chronic neurodegenerative disease that interferes with cerebral function by progressively impairing memory, thinking and learning through the continuous depletion of neurons. Although its etiopathogenesis remains uncertain, recent literature endorses the hypothesis that probiotic, prebiotic and synbiotic supplementation alters AD-like symptoms and improves many of its associated disease biomarkers. Alternatively, a dysfunctional microbiota impairs the gut epithelial barrier by inducing chronic gastric inflammation, culminating in neuroinflammation and accelerating AD progression. The findings in this review suggest that probiotics, prebiotics or synbiotics have potential as novel biological prophylactics in treatment of AD, due to their anti-inflammatory and antioxidant properties, their ability to improve cognition and metabolic activity, as well as their capacity of producing essential metabolites for gut and brain barrier permeability.

## Introduction

Alzheimer’s disease (AD) is a chronic neurodegenerative disorder characterized by a progressive decline in cognitive function, eventually culminating in a loss of memory, thinking and reasoning. AD has become a pandemic issue due to its widespread distribution, affecting over 50 million people worldwide and a projected 152 million people by 2050 (Brookmeyer et al., 2007). Such an increase in prevalence has led to a growing public demand for effective AD therapeutics. Several candidate drugs have been proposed targeting a variety of mechanisms, including the formation of extracellular aggregations of amyloid beta (Aβ) plaques, neurofibrillary tangles (NFTs) composed of hyperphosphorylated tau proteins, bacterial infection, neuroinflammation or oxidative stress, but most have proven unsuccessful or unreliable in averting disease progression (Schneider, 2016). Recent literature describes the complex nature of AD as a multi-faceted disorder (Wang et al., 2019). Indeed, environmental and genetic factors have been documented to play a role in AD onset, suggesting that the design of a multi-targeted therapy may offer a more robust approach for treating AD. Supplementation with prebiotics, probiotics and synbiotics (a combination of pre and probiotic ingredients) meets the criteria for such a holistic therapeutic strategy, due to the widespread effects of these biopharmaceuticals on both gut microbiota and host physiology. Much recent work has illustrated the diverse benefits of pre/probiotic therapy in autoimmune, inflammatory and allergic disorders, benefits that may in fact extend into the realm of neurodegenerative pathology.

Until recently, bacteria have been largely perceived as exclusively pathogenic (Ricci et al., 2015), a view that manifested as a pervasive “us-versus-them” mentality for the majority of the twentieth century. “Cleansing” humans of bacteria was a doctrine that would only be rebutted in the 1990s, when the dangers of overprescribing antibiotic compounds were unveiled and the benefits of probiotic bacteria surfaced (Guy Leblanc et al., 2010; Villanueva-Millán et al., 2015). This revolution was shortly followed by attempts to regulate one’s commensal bacterial population, a process labeled as microbiome modulation. The human microbiome is composed of non-pathogenic microorganisms including over 10,000 different species (Mittelman and Burstein, 2019), whose symbiotic relationships to the host are quintessential in maintaining digestive, respiratory, hepatic, and immune health (Ushakova et al., 2015; Nursalim et al., 2016; Gurevich et al., 2018; Wang and Ji, 2019). Consistent with this notion, several studies note that perturbation of a healthy gut microbiota is linked to a variety of pathological states, including neurodegeneration, suggesting a role for microbiome modulation in restoring patient health. Although there is insufficient evidence to posit a curative role for pre, pro and synbiotics in treatment of AD, the lack of success in AD drug discovery has motivated much investigation into their use as dietary supplements in the mitigation of AD-like symptoms. Hence, several proposed modes of action for the effects of pre/probiotics on neurodegeneration will be outlined in this review.

Several studies have established the correlation between disruption of the human microbiome and various biomarkers of neurodegenerative disorders, especially AD. Although probiotics have become well-known for their contribution to gastrointestinal (GI) health, much remains unknown regarding their full therapeutic potential in correcting such a pathology-related degradation to the microbiome ecosystem and thus altering the disease state. In fact, a probiotic-induced balanced microbiome has shown the ability to improve AD symptoms through various mechanisms. These outcomes include antioxidants production and subsequent neutralization of reactive oxygen species (ROS) in the GI tract, downregulation of cytokine-induced proinflammatory cascades, and the strengthening of epithelial barrier function to protect the enteric nervous system (ENS) from potentially toxic compounds (Plaza-Díaz et al., 2017; Wang et al., 2017; Chelakkot et al., 2018). The latter two mechanisms depend on the microbiome’s ability to interact with the central and ENSs, in a bilateral communication channel constituting what is referred to as the microbiota gut-brain axis (MGBA). The ENS populates the second most neurons in the entire body and is responsible for some fundamental functions of the GI tract such as motility, mucosal secretions, GI angiogenesis, and maintenance of blood flow homeostasis (Chandrasekharan et al., 2019). Transmission of information between the GI tract and the brain, is largely mediated by the vagal nerve through various neurotransmitters such as serotonin, dopamine, GABA, and glutamate (Solari et al., 2017; Choe et al., 2019).

The MGBA involves a myriad of biochemical, neurological and endocrine pathways and has been postulated as a biological underpinning of many misunderstood neurological disorders, especially AD. This is in large part due to how the fate of the blood-brain barrier (BBB) is intertwined with both host gut microbiota and AD pathology (Gilbert et al., 2013; Hyland and Stanton, 2016; Skonieczna-Żydecka et al., 2018; Gomez-Eguilaz et al., 2019). The BBB is a tightly regulated partition composed of endothelial cells linked paracellularly by tight junctions (TJs) whose purpose is to dynamically restrict undesired and harmful bloodborne substances from the brain parenchyma. A tightly regulated BBB is required for proper brain function, and disruption of BBB integrity is a hallmark of neurodegenerative pathology (Zenaro et al., 2017). Such disruption can be initiated by a plethora of mechanisms, one of which being an injured gut microbiota. Thus, optimizing one’s microbiota through the supplementation of prebiotics, probiotics or synbiotics can prompt a flourishing gut, but above all, a healthy brain.

Prebiotics are dietary fibers that cannot be digested by the human digestive system thereby acting as food to the gut microbiota. While prebiotics interact minimally with the host, their regular consumption leads to the establishment of a balanced commensal microbiota. Specifically, prebiotics act as fermentation substrates for short-chain fatty acid (SCFA)-producing probiotic genera such as *Bifidobacteria* and *Lactobacilli*, leading to an increase of the anti-inflammatory metabolites acetate, propionate and butyrate. SCFAs have a direct impact on the host’s innate and adaptive immune system, by regulating T cell differentiation through the activation of gut epithelium G-protein coupled receptors (GPCRs). T cells are antigen-recognizing white blood cells that play a major role in the regulation of tissue inflammation. SCFAs can promote the differentiation of naïve CD4 + T cells into its Th1 and Th17 phenotypes, leading to the production of anti-inflammatory cytokines such as IL-10 (Kim et al., 2014; Sun et al., 2018);. Additionally, SCFAs can bind to metabolite-specific cell receptors on gut-associated lymphoid tissue, inhibiting the production of pro-inflammatory cytokines (Mirmonsef et al., 2012). The combination of these effects suggests that the upregulation of SCFA-producing bacteria could be a key mode of action by which prebiotics, probiotics and synbiotics may affect AD patients, by attenuating the chronic systemic inflammation that plays an instrumental role in AD pathogenesis.

Despite the belief that prebiotics and probiotics only affect gut health, recent studies elucidating their potential impacts on the MGBA have advanced these microbiome-modulating bioactives into the field of neurodegenerative diseases and neurocognitive disorders. There have been several biotherapeutics investigated in clinical trials since; many advancing to phases 2–3 human studies. These include *AB-2004* from Axial Biotherapeutics targeting Autism Spectrum Disorder (Biotherapeutics, 2019) and *ENT-01* from Enterin Inc., targeting the dysfunctional gut microbiota and constipation in Parkinson’s Disease patients (Enterin, 2019). However, only five proposed drugs for AD have been clinically approved by the U.S. Food and Drug Administration, none of which are microbiome modulating biotherapeutics. Overall, probiotics, prebiotics and synbiotics have not yet permeated the realm of pharmaceutical products. However, considering their well-documented benefits and their ability to target multiple symptoms in the confines of AD pathology, a biotherapeutic composed of probiotics, prebiotics or both is imminent.

**FIGURE 1:**
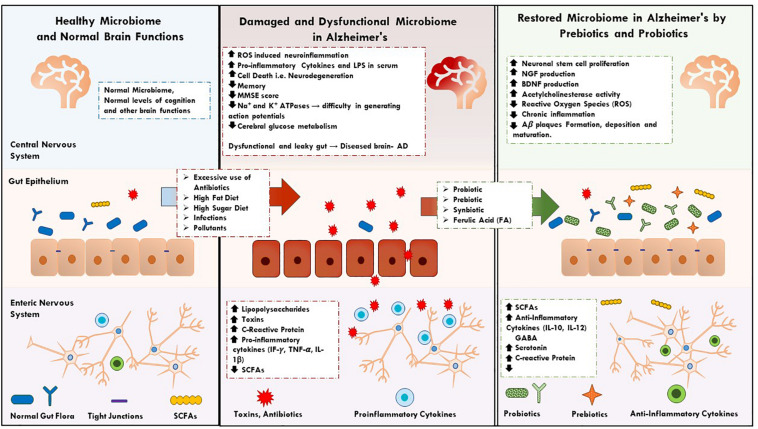
It describes the key factors and events in normal, dysfunction human microbiome and restoration of microbiome in AD by prebiotics, synbiotics, and probiotics supplementations.

Although literature attempting to grasp AD’s etiology is abundant, little is known regarding what causes its onset and perpetuation. Studies in this review suggest that prebiotics, probiotics, and synbiotics impact several symptoms and biomarkers of AD, cementing their potential as future biotherapeutics for the disease. The role of a healthy microbiome in the prevention and mitigation of AD, and evidence supporting such a novel microbiome-based therapeutic strategy will be further discussed in this review (Figure 1).

## Probiotics as Alzheimer’s Disease Health Supplement

While AD’s etiopathogenesis remains misunderstood, recurring indicators including increased oxidative stress, inflammation, and severe cognitive impairment are the common indices for diagnosis. Probiotics have demonstrated significant potential in the deceleration of Alzheimer’s disease progression when consumed as a lone supplement. Indeed, their clinically tested anti-inflammatory properties, antioxidant properties as well as their ability to increase a patient’s cognitive aptitude have become the main driving factor behind investigation of overall therapeutic utility.

### The Role of the Dysfunctional Microbiota in AD Pathology

The gut microbiota maintains a stable gastrointestinal environment assisting in many human digestive, immune and cognitive functions. Like most homeostatic systems, if stability is disrupted beyond regulatory limits, the cascading effects can be dire. A perturbation in the microbial ecosystem is commonly referred to as dysbiosis, or simply a dysfunctional microbiota. As will be discussed later in the review, probiotic supplementation, whether consumed alone or in conjunction with prebiotics or foods, have beneficial effects on AD related symptoms. However, this first warrants a discussion on how a dysbiotic microbiome influences and contributes to the progression of AD pathogenesis.

An increasingly prominent mode of action by which gut bacteria can play a role in central nervous system deficiency involves the altered metabolism of bile acids (BAs). BAs are hydroxylated steroids synthesized by the liver to assist in cholesterol metabolism. Primary BAs such as cholic acid and chenodeoxycholic are deconjugated by gut bacteria to form secondary bile salts, such as deoxycholate and litocholate, which are ultimately reabsorbed in the ileum and colon and redirected to the liver for recycling into the BA pool (Ridlon et al., 2006). The process of primary BA metabolism by gut bacteria has recently emerged as an indicator for gut dysbiosis. Indeed, elevated concentrations of secondary BAs are observed in stool or serum samples when anaerobic pathogenic bacteria are disproportionately prevalent in the large intestine. Notably, the group of Nho et al., sampled the serum levels of 15 BAs and their conjugate counterparts in 1,464 subjects at various levels of cognitive function and AD progression (normal cognition; early mild cognitive impairment, MCI; late MCI and AD). They noticed substantially lower levels of cholic acid and an increased concentration of its microbially metabolized counterpart, deoxycholic acid, suggesting that an increase in BA dihydroxylation by gut bacteria could be associated with cognitive impairment. In a follow-up study, the group examined and compared BA profiles of 566 serum samples and 111 post-mortem brain tissue samples from cognitively impaired patients. They found similar ratios of various primary and secondary BAs present both sample types, suggesting a potential mechanism for BA translocation to the brain from systemic circulation. The authors further postulated that reduction in cognitive function may be partially attributed to the cytotoxic properties of deoxycholic acid, which is capable of disrupting the blood-brain barrier (BBB) and penetrating into brain tissue. In fact, several studies suggest that an elevation in serum BA levels may induce an increase in BBB permeability by way of tight junctions disruption (Quinn et al., 2014). Interestingly, elevated bile acids have been also been shown to modulate TJ permeability at the level of the gastrointestinal epithelium (Raimondi et al., 2008; Stenman et al., 2012), implicating microbiome-mediated luminal BA homeostasis in determining peripheral plasma concentrations and systemic effects of these pleiotropic metabolites. These studies indicate that although commensal bacteria may positively affect cognition, a dysbiotic gut microbiota can be equally detrimental to AD patients via indirect inflection of BA metabolism. Albeit speculative by the authors, there is a growing body of evidence in support of this hypothesis, further strengthening the theoretical link between bacterially produced bioactive molecules, the BBB and cognitive function.

In the realm of beneficial bacterial metabolites, SCFAs have garnered much interest within the past decade for their valuable biotherapeutic properties. The SCFAs acetate, butyrate and propionate are bacterial fermentation by-products that are known to enhance barrier function of the gut epithelium separating the ENS from the luminal environment (Lawson, 1990). Gut epithelial integrity is tightly controlled by intercellular tight-junction proteins, whose expression and composition can be enhanced by SCFAs to strengthen the gut barrier. By contrast, weak epithelial tight-junction interactions increase the permeability of the gut lining to luminal endotoxins, allowing them to enter circulation. This in turn can induce a systemic pro-inflammatory response, eventually culminating in chronic neuroinflammation. It has been shown that germ-free (GF) mice display a decrease in tight junction (TJ) protein levels, accompanied by clear deficiencies in non-spatial and memory-based exercises, as well as a reduced level of BDNF and a decreased expression of N-methyl-D-aspartate receptor (NMDAr) mRNA in the hippocampal and cortex regions (Hu et al., 2016). Both BDNF and NMDAr play a critical role in neuroplasticity which has proven to be a major indicator of AD etiology (Wang and Reddy, 2017). Interestingly, these deficits were remediated when the same mice were transplanted fecal samples from balanced-microbiota mice, indicating an important role in regulation of the enteric-immune interface.

Lipopolysaccharide (LPS) is a common component of Gram-negative bacteria cell wall, comprising an important category of endotoxins that can induce a pro-inflammatory response via translocation into the bloodstream. AD patients experienced a threefold increase in serum LPS concentration compared to their healthy counterparts (Zhan et al., 2018), further consolidating the hypothesis that a permeable gut epithelial barrier contributes to AD onset. High levels of LPS were also observed in the hippocampal and temporal lobes of AD patients, suggesting that gut-derived inflammation might be communicated to the brain via the disruption of the BBB and lead to a prolonged neuroinflammatory state. Indeed, recent studies show that LPS can cross the BBB via interaction with the constitutively expressed CNS receptors CD14 and TLR-4, further eliciting an innate immune response in the brain with subsequent neuroinflammation and cell death (Vargas-Caraveo et al., 2017). These studies suggest that the strengthening of barrier function, whether at the level of brain or gut, might be a potential avenue for AD prophylactics (Braniste et al., 2014; Zhao et al., 2017). Pathogenic bacterial strains such as *Escherichia coli* spp., *Bacillus subtilis* and *Staphylococcus aureus* are prevalent producers of Aβ and LPS (Pistollato et al., 2016), and have the tendency to proliferate and compete against commensal bacteria such that the microbiota ecosystem spirals into a dysbiotic state. This initiates a cascade of processes in which a dysfunctional microbiota engenders accumulation of Aβ in the gut, then in the brain microvasculature, which subsequently induces microglial activation, a sustained immune response, and prolonged neuroinflammation. On the other hand, several probiotic strains have been shown to enhance gut barrier function through the production of SCFAs and upregulation of tight junction expression. Such strains include *Lactobacillus plantarum* WCFS1, *E. coli Nissle* and *Bifidobacterium infantis* spp. (Kowalski and Mulak, 2019). Considering the large amounts of Aβ and endotoxin present in the gut microbial milieu and their pro-inflammatory properties, maintaining a secure barrier between the GI tract, the vascular system and the BBB through consumption of probiotics is critical to host health and immune system homeostasis.

### Probiotics as Anti-inflammatory Agents in AD

The immune system and the bacteria populating the gut have a unique but fragile relationship, in which immune cells must differentiate helpful, innate bacteria from pathogenic, foreign bacteria. This tightly regulated identification mechanism, if faulty, can lead to unnecessary immune responses, prompting low-grade chronic inflammation (Mafra et al., 2014). The identification of innate versus foreign bodies in the intestine is initiated by intestinal epithelial cells. These cells are responsible for producing the adequate macrophage phenotype (M1 or M2), based on the bacterial antigens present at the bacteria’s cell surface and within its microenvironment. Examples of antigens that can be commonly encountered on and around the microorganism are lipopolysaccharides (LPS), peptidoglycan and flagellin. Although these bacterially derived molecular patterns are considered innate to intestinal epithelial cells, translocation of endotoxins such as LPS into vasculature will upregulate local production of pro-inflammatory cytokines such as tumor necrosis factor-alpha (TNF-α), interleukin (IL)-6 and IL-1, which can ultimately lead to septic shock (Shapira et al., 1996). Translocation of LPS occurs when the epithelial barrier function is compromised, either by a reduction in tight junction proteins connecting epithelial cells or by the depletion of the epithelial mucosa. This can lead to a prolonged state of inflammation in the gut as well as in the brain, given that endotoxins can migrate to and interact with the BBB. Probiotics can considerably diminish systemic inflammation and LPS translocation by several mechanisms, all of which will be discussed in the following section.

A plausible mode of action whereby the gut microbiota affects brain amyloidosis was investigated by Cattaneo et al. (2017) in a study assessing the pro- and anti-inflammatory cytokine activity of several gut microbiota taxa in cognitively impaired patients. To do so, the patients’ microbiota composition in stool samples was scoured for candidate gut microbial taxa, and serum levels of inflammatory cytokines were quantified. The bacteria to be quantified were selected due to their established pro-inflammatory properties (*Escherichia/Shigella* and *Pseudomonas aeruginosa*) and anti-inflammatory properties (*Eubacterium rectale, Eubacterium hallii, Faecalibacterium prausnitzii*, and *Bacteroides fragilis*). Peripheral inflammatory markers hypothesized to be involved in the neuroinflammatory pathogenesis of AD were also quantified, in an attempt to correlate a pro- (CXCL2, CXCL10, IL-1β, IL-6, IL-18, IL-8, inflammasome complex NLRP3), TNF-α) or anti-inflammatory (IL-4, IL-10, IL-13) cytokine expression to the gut microbiota composition. Of the abovementioned bacterial strains, only an increase in *Escherichia/Shigella* and a decrease in *Eubacterium rectale* statistically correlated with changes in the pro- and anti-inflammatory cytokine profiles of the cognitively impaired and amyloid positive patients, suggesting that these strains might contribute to the pro-inflammatory state seen in AD patients. Additionally, the patient’s showed an increased level of the pro-inflammatory cytokines IL-6, CXCL2, NLRP3, and IL-1β, as well as a decreased level of the anti-inflammatory cytokine IL-10. Consistently, patients displaying increased proportions of *Escherichia/Shigella* also displayed increased levels of IL-1β, CXCL2, and NLRP3, while an abundance of *E. rectale* demonstrated a negative interdependence with the same pro-inflammatory cytokines and a positive interdependence with IL-10. These findings support the hypothesis that the gut microbiota composition could initiate, worsen or alleviate peripheral inflammation experienced in AD patients. However, to establish a causal relationship between the gut microbiota and amyloidosis requires more insight on how chronic systemic inflammation can translate to neurodegeneration, a key conjecture demystified in section “*Probiotics and Cognition.*”

**FIGURE 2:**
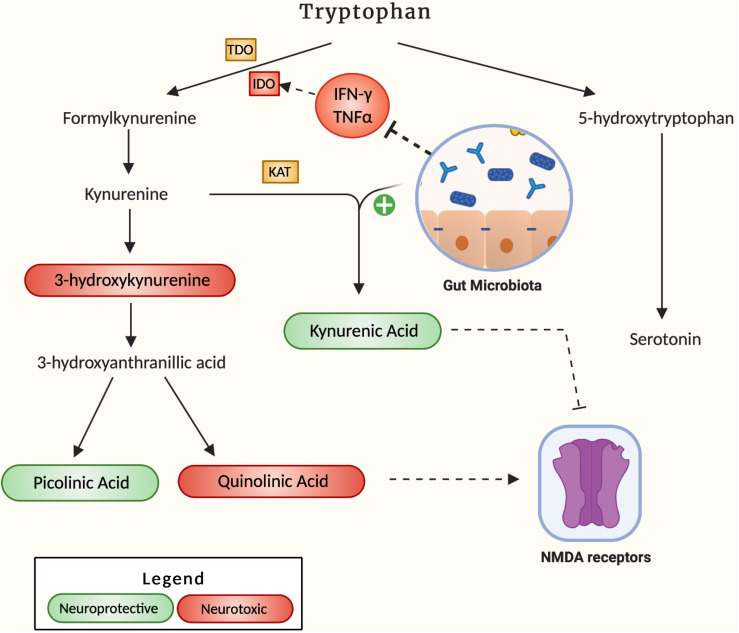
It describes how the gut microbiota affects the kynurenine pathway. Neuroprotective by-products are labeled green, and neurotoxic by-products are labeled red. The mechanism by which the gut microbiota increases the production of KA is not specified in literature.

A hypothesized pathway by which neuroinflammation can develop in the brain of AD patients is through the dysregulation of the kynurenine pathway namely the process through which 95% of the body’s tryptophan is metabolized (Figure 2). The downstream products of this pathway comprise a set of 4 key metabolites, 3-hydroxykynurenine (3-HK), quinolinic acid (QA), kynurenic acid (KA) and picolinic acid, that play a pivotal role in energy production and neuroplasticity (Savitz, 2020). However, if present in irregular proportions, the metabolites 3-HK and QA can become neurotoxic, leading to microglia activation and cell death (Lugo-Huitrón et al., 2013). Due to its potential neurotoxicity, the kynurenine pathway has been investigated for its potential role in the pathogenesis of AD. In fact, indoleamine 2,3-dioxygenase 1 (IDO-1), a subset of the key tryptophan-metabolizing enzymes in the kynurenine pathway, is stimulated by the pro-inflammatory cytokine IFN-γ and has been shown to colocalize with Aβ plaques accordingly (Desbonnet et al., 2008). In addition, QA, a downstream product of kynurenine, is a known NMDA receptor antagonist, which can ultimately result in glutamate accumulation in the receptor’s periphery, excitotoxicity and eventual widespread brain tissue damage. QA has been found to accumulate in the hippocampus of AD patients (Guillemin, 2012), suggesting a role for this process in the hippocampal atrophy that often accompanies AD progression. Widner et al., 2000 also noted an elevated kynurenine to tryptophan ratio in AD patient’s serum compared to controls, indicative of an elevated rate of tryptophan breakdown. An accumulation of the neurotoxic by-products resulting from this accelerated degradation positively correlated to an increase in CRP, a key inflammatory marker (Leblhuber et al., 2018). Indeed, the induced pro-inflammatory response from downstream IDO metabolites suggests that tryptophan degradation may be a key contributor to AD pathogenesis, and that favoring alternative pathways converting this precursor to serotonin may alleviate inflammatory tone. In this regard, Valladares et al., 2013 were able to decrease endogenous IDO levels through administration of *Lactobacillus johnsonii* to germ-free mice, accompanied by decreased serum levels of kynurenine and a 1.4-fold increase in serotonin. Together these observations suggest that gut microbes exert a potent effect on the metabolic fate of tryptophan, promoting serotonin formation from tryptophan over kynurenine pathway degradation. Another study by Desbonnet et al. (2008) reported the anti-inflammatory effects of *Bifidobacterium infantis* by measuring tryptophan and kynurenic acid levels in probiotic-treated rats when compared to controls. They observed increased plasma concentration of tryptophan complemented by increased serum kynurenic acid, suggesting a decrease in the associated pro-inflammatory effects of the pathway. Although the upside of probiotics as anti-inflammatory agents is apparent, further humanized studies are required to establish probiotics as therapeutics for neurodegenerative diseases. Regardless, their effects are well-documented, and their potential remains to be uncovered.

### Probiotics as Antioxidants in AD

In the 19th century, atomic energy plants were revered for their incredible energy production, neglecting the potentially catastrophic ramifications of the dangerous toxic waste associated with this process. Analogously, the human body’s energy-producing mechanism is incredibly efficient, but a by-product of the process is free radicals in the form of ROS. These entities include oxygen atoms lacking one electron for the completion of their octet, making them potent oxidizers that can mediate nucleic acid breakage, enzyme inactivation, lipid peroxidation and polysaccharide depolymerization (Wang, 1998). Ultimately, ROS can cause massive damage to intracellular biomolecules, culminating in cell death. To neutralize free radical oxygens the body produces some endogenous antioxidants, such as glutathione (Ruan et al., 1997). However, reliance on glutathione’s antioxidative properties may lead to its rapid depletion and a sustained pro-inflammatory state: a potent risk factor for AD onset. It follows that administration of exogenous antioxidants may attenuate the pro-inflammatory response resulting from excessive levels of ROS, in turn limiting chronic neuroinflammation (McGeer and McGeer, 2001; Janciauskiene et al., 2002).

In this regard, many antioxidant compounds have arisen as candidate supplements to bolster endogenous ROS scavenging capacity. For centuries, Chinese medicine prescribed plant mixtures containing an abundant amount of Ferulic Acid (FA) due to its antioxidative and anti-inflammatory properties (Nabavi et al., 2015). FA is a phenolic compound which drives neuronal stem cell proliferation and reverses chronic inflammation in mice by increasing production of nerve growth factor (NGF) and brain-derived neurotrophic factor (BDNF), two neuropeptides central to neuronal regeneration (Lindsay, 1988; Meng et al., 2018). In addition to dietary sources, FA is produced by probiotic bacteria via a group of enzymes known as FA esterases. Probiotic species such as *Lactobacillus plantarum* NCIMB 8826, *Lactobacillus fermentum* NCIMB 5221 and *Bifidum animalis* have demonstrated the ability of producing the antioxidant in large quantities (Westfall et al., 2017). Indeed, FA production in probiotics has garnered a substantial amount of attention for its therapeutic effect against AD, due to its ability to target multiple facets of the disease. For example, pre-treatment with FA has been shown to reverse neuroinflammation in transgenic AD-mice, as well as decrease hippocampal and cortical levels of Aβ fibrils when compared to controls lacking the probiotic-produced phenolic compound (Ono et al., 2005). A hypothesized mechanism of action involves the partial insertion of FA between Aβ peptides, mediated by presence of hydroxyl and carboxyl moieties that permit hydrogen bond formation between these peptides. Overall, FA-producing probiotics have shown to not only prevent the development of AD through scavenging ROS, but could also inhibit the formation, deposition and maturation of amyloid-beta (Aβ) in a dose-dependent manner. Approaching AD pathogenesis in this multi-faceted manner may offer a resolution to the shortcomings of many candidate AD pharmaceuticals explored to date, and potentially become the staple therapeutic approach for future drug discovery.

A secondary proposed mechanism of action for probiotic-derived antioxidants revolves around the sirtuin-1 (SIRT1) protein deacetylase, which has documented neuroprotective benefits resulting from the regulation of several antioxidant genes. An investigation by Bonfili et al. (2018) explored the ability of the probiotic formulation SLAB51 (*Streptococcus thermophilus, Bifidobacterium longum, B. breve, B. infantis, Lactobacillus acidophilus, L. plantarum, L. paracasei, L. delbrueckii subsp. bulgaricus, L. brevis*) to counteract early oxidative stress in AD-induced mice, honing in on a potential molecular mechanism of action for its antioxidative activity. They found expression of SIRT1 was substantially decreased in the brains of untreated transgenic 3xTg-AD-mice, whereas the activity and expression of SIRT1 was restored by supplementation of the probiotic mixture SLAB51. Increased activity of SIRT1 was also confirmed based on an increase in deacetylation of its primary substrate, retinoic acid receptor-β (RARβ). Deacetylation of the latter stimulated the production of ADAM-10, an α-secretase that cleaves APP in the non-amyloidogenic pathway, which led to decreased formation of Aβ. Furthermore, an increase in SIRT1 activity by the lactic acid and bifidobacterial supplementation reduced the acetylation of p53 protein, promoting cell survival under stressful conditions by suppressing apoptotic pathways. Finally, the scientists measured the functionality of redox enzymes and quantified the presence of cellular oxidation markers so as to evaluate the probiotic mixture’s ability to activate the SIRT1 pathway and promote antioxidative effect. Older AD mice showed a decrease in the antioxidant enzymes GPx and catalase, whereas administration of SLAB51 mitigated this effect, thus alleviating the oxidative stress-causing impairments. Similar findings have been recapitulated in humans: decreased levels of SIRT1 were observed in AD patients resulting in accumulation of Aβ and hyperphosphorylated tau proteins in the cortex, and an increase in cerebral ROS leading to neuronal apoptosis and necrosis (Julien et al., 2009).

### Probiotics Improve Cognition

Apart from the physiological symptoms of AD patients also experience a progressive loss of memory and cognitive function, in part due to Aβ-induced neuronal instability. A proposed mode of action by which the gut microbiota affects cognition involves the chronic activation of glial cells due to increased oxidative stress and inflammatory tone. Glial cells act as the primary constituents of the brain’s immune system, and are key contributors to the regulation of neuronal homeostasis and survival (Mandrekar-Colucci and Landreth, 2010). In the scenario of bacterial or viral infection, microglia enter an activated state whereby their objective becomes to phagocytize the foreign body. Once the threat is removed, their state is returned to its basal phenotype. In AD however, the BBB and gut-epithelial barrier is compromised, allowing for translocation of toxic compounds such as foodborne toxins, LPS and pathogens. This leads to the continuous activation of microglia, which persist in their phagocytic state, and constantly release pro-inflammatory cytokines to mitigate this sustained state of perceived infection. Ultimately, excessive microglial production of cytokines such as IL-1β, IL-6, and most importantly TNFα, prompt neuronal cell death. Indeed, memory loss, a common symptom of AD, corresponds to a neuronal loss in the hippocampal area of the brain (Katsumoto et al., 2018). Alternatively, loss of neurons in other regions of the frontal cortex leads to the progressive decline in language, reasoning and behavior. Pathways by which the gut microbiota may affect neuronal cell death are diverse, and studies investigating these pathways will be outlined in the following discussion.

The group of Chu et al. recently proposed a mechanism by which the gut microbiota could alter cognitive function, through a specific sub-set of learning termed extinction learning. Extinction learning is a common psychoanalytical method related to Pavlovian fear conditioning, where a conditioned stimulus, such as tone, is coupled with an unconditioned stimulus, usually an electric shock. This incites mice to form new memories associating the stimuli pairs to a fearful experience, which can be monitored by bulk-RNA sequencing and snRNA sequencing of the medial prefrontal cortex or the frontal association cortex over time. They observed a decreased extinction learning in antibiotic-treated adult mice (ABX) mice compared to untreated controls, that was related to the remodeling of dendritic spines and a reduced neuroplasticity in association and signal-encoding neurons of the medial prefrontal cortex. Microglia in ABX mice were also significantly underdeveloped, further aggravating the reduction in extinction learning of mice by obstructing dendritic spine formation. Gnotobiotic mice at different developmental stages (birth, weaning, adult) were then colonized with a diverse microbiota and the deficiency in memory formation was re-examined. Although the adult and weaning mice did not demonstrate changes in their defective extinction learning, the mice colonized after birth with the microbiota of their surrogate mothers displayed extinction behavior comparable to the control mice. This led to the authors’ conjecture that learning-related plasticity may be influenced by a diverse gut microbiota during the mice’s developmental period, and that bacterially produced metabolites may be the driving mechanism for an effective memory-formation process. Indeed, four candidate metabolites were significantly altered in cerebrospinal fluid (CSF), serum and fecal samples of adult ABX, control and restored-microbiota mice. Through unbiased comparative analysis of MS datasets, it was determined that phenyl sulfate, pyrocatechol sulfate, 3-(3-sulfooxyphenyl) propanoic acid, and indoxyl sulfate were all substantially reduced in CSF, serum and fecal samples of the ABX mice compared to control and restored-microbiota mice. This evidence supports novel explanation for the altered cognitive function in germ free mice, suggesting that bacteria-derived metabolites play a role in neuronal activity and behavior.

Another route by which the gut microbiota may impact cognition is through modulation of global cortical and hippocampal activity. Recent studies suggest that Aβ plaques formed in the frontal cortex and cerebellum regions led to a decrease in Na + and K + ion concentration in neurons, which in turn led to a diminished level of Na + and K + -ATPase transporters at the cell periphery (Kairane et al., 2002; Mallikarjuna et al., 2016). These neuronal membrane-bound proteins regulate the proton gradient across membranes and are responsible for maintaining the cell’s ability to generate action potentials. Thus, a significant decrease in ATPases may contribute to the initiation of AD pathogenesis, which would be further exacerbated by oxidative stress and the ensuing decrease in cerebral energy metabolism. To investigate said hypothesis, D-galactose was administered to mice to mimic the inhibited neuronal metabolism, mitochondrial dysfunction and oxidative stress observed in AD patients (Chiroma et al., 2018). D-galactose is a reducing sugar which inhibits the production of NGFs, Acetyl Choline and a myriad of associated proteins when administered at high doses (Luo and Xiao, 2004). Continual supplementation of D-galactose in mice resulted in a 20% decrease in total membrane bound ATPases, inducing AD-like symptoms. Treatment with *Lactobacillus plantarum* MTCC1325 restored the ATPase enzyme levels for all subjects (*n* = 30) in the cerebral cortex and hippocampus regions. Coincidently, AD-induced mice experienced a significant reduction in Na + and K + ATPase levels in both the cerebral cortex and hippocampus. Post-*Lb plantarum* supplementation corresponded to a near-complete recovery in ATPase activity in both cerebral regions of the AD mice (Chiroma et al., 2018). These results demonstrate the promising restorative benefits of probiotics in the augmentation of neuronal function, through restoration of membrane ionic gradient in brain areas associated with memory, language, reasoning and association.

Although the more common areas of research when investigating AD pathogenesis involves biomarkers of oxidative stress and inflammation (Gahtan and Overmier, 1999; Cai and Yan, 2007; Galasko and Montine, 2010), dysfunctional cerebral glucose metabolism can easily be quantified through insulin levels and has also been recognized as a key pointer of early onset of AD (Sims, 1990; Kuehn, 2020). Glucose hypometabolism and insulin signaling are responsible for regulating energy levels in the brain. Considering the organ’s large demand in energy, faulty glucose metabolism may trigger a cascade of disruptive processes such as insulin resistance, further inducing neurodegeneration (Craft, 2007). A recent randomized, double-blind and controlled clinical trial involving 60 AD patients was conducted to investigate the effect of *Lactobacillus acidophilus, Lactobacillus casei*, *Bifidobacterium bifidum, and Lactobacillus fermentum* on AD-related biomarkers and symptoms (Akbari et al., 2016). A Mini-mental state exam (MMSE), homeostasis model assessment of insulin resistance, as well as a quantification of the inflammation biomarker C-reactive protein (CRP) were analyzed pre- and post-intervention. The MMSE is a commonly used exam to determine an AD patient’s cognitive function by testing their sense of time, orientation, memory, space and sight. In medicine, it is used to screen for neurodegeneration but in a clinical environment, it is employed to qualify the subjects’ progression or regression in cognitive ability. In this study, the MMSE score of the patients treated with the probiotic mixture experienced a significant increase (+27.9% ± 8.07) relative to their untreated counterpart (−5.03% ± 3.00). Likewise, probiotic treatment had a considerable impact on serum CRP levels in AD patients (−17.61% ± 3.70), compared to controls (+45.26% ± 3.50) who experienced a significant increase in the biomarker level, results that are consistent with the aforementioned anti-inflammatory properties of probiotics. Finally, treatment with *Lb. acidophilus*, *Lb. casei*, *B. Bifidum* and *Lb. fermentum* decelerated the rate at which insulin resistance manifested in the brain of AD patients (+28.84% ± 13.34) compared to controls (+76.95% ± 24.60) (Akbari et al., 2016). Thus, supplementation with probiotics appears to not only slow down the progression of neurodegeneration but re-establish previously disrupted processes in the brain via the MGBA. Mounting clinical evidence support the hypothesis that probiotics positively affect AD patients, in a multi-targeted approach so as to restore the patient’s cognitive aptitude.

## Probiotics in Combination With Prebiotics or Foods as AD Health Supplements

Although administration of prebiotics or probiotics alone has shown promise as prophylactics for AD, the bioavailability of prebiotic fibers as well as the colonization potential of probiotic bacteria present barriers to their overall efficacity and potency. Indeed, the bioavailability and bioactivity of prebiotics heavily depends on gut microbiota diversity, as selective fermentation of different prebiotic fibers by various strains may influence composition and size of the downstream metabolite pool (Espín et al., 2017). However, a single strain of probiotic bacteria often has limited impact on gut composition, and is seldom considered a therapeutic in isolation due to the high variability in gut-mucosal colonization rates and lack of causality of results (Zmora et al., 2018). However, when supplied with adequate dietary/prebiotic substrate, several bacterial strains can work synergistically in a process termed cross-feeding, whereby the product of one strain’s metabolism may be utilised in the nutrition of another (Smith et al., 2019). This interdependence is exploited in synbiotic formulations, wherein prebiotics supply the substrates for the proliferation of beneficial bacteria and these bacteria enhance bioavailability of prebiotic-derived anti-inflammatory and antioxidant metabolites. As will be catalogued in the following section, the benefits of prebiotics and synbiotics are numerous. The comparative effects of synbiotic formulations, prebiotics and probiotics are further characterized in Table 1.

**TABLE 1:** Summarizing documented effects of Pre, Pro, and Synbiotic formulations on AD patients and animal models of AD.

**Probiotics**	**Subject/Model**	**Effect**	**Source**
*Lactobacillus johnsonii*	GF mice	Decreased serum levels of kynurenine (a by-product of tryptophan) and increased serotonin levels by 140%. Suggesting that the probiotic promoted the serotonergic pathway over the neurotoxic kynurenine pathway	Valladares et al., 2013
*Bifidobacterium infantis*	Sprague-Dawley rats	Plasma concentration of tryptophan increased whereas the concentration of kynurenic acid decreased, followed by a decrease in the pro-inflammatory response from the kynurenine pathway	Desbonnet et al., 2008
*Lactobacillus fermentum* NCIMB 5221	Male APPswe and PS1ΔE9 mutant transgenic mice	Produces Ferulic acid, a natural antioxidant and anti-inflammatory agent. Pretreatment of FA to Aβ induced mice resulted in the mitigation of neuroinflammation Aβ fibril formation, and restored memory deficits	Westfall et al., 2017; Mori et al., 2019
*SLAB51 (Streptococcus thermophilus, Bifidobacterium longum, B. breve, B. infantis, Lactobacillus acidophilus, L. plantarum, L. paracasei, L. delbrueckii subsp. bulgaricus, L. brevis)*	3 × Tg-AD-mice	Restoration of SIRT1 protein deacetylase activity. Stimulation of the ADAM-10 α-secretase, cleaving APP in a non-amyloidogenic pathway, inhibiting the formation of Aβ. Decreased acetylation of p53 protein, promoting neuronal cell survival	Bonfili et al., 2018
*Lactobacillus plantarum* MTCC1325	D-galactose induced AD-mice	Restored overall ATPase enzyme levels for all subjects in the cerebral cortex (CC) and hippocampus (HP) regions. Recovered Na+ and K+ ATPases in the CC and HP, leading to the regulation of membrane ionic gradient for an adequate neuronal activity	Nimgampalle and Yellamma, 2017; Chiroma et al., 2018
*Lactobacillus acidophilus, Lactobacillus casei, Bifidobacterium bifidum*, and *Lactobacillus fermentum*	Human AD patients	MMSE score increasd relative to the AD patients, significant decrease in CRP levels and a decelerated rate at which insulin resistance affects the brain and subseqquent neuronal cell death	Akbari et al., 2016
*Lactobacillus plantarum WCFS1, E. coli Nissle* and *Bifidobacterium infantis* spp.	–	Proficient in producing SCFAs such as butyrate, propionate and acetate. The former also has BA metabolism properties; a mechanism important in reducing the amount of cytotoxic secondary BAs in the brain	Kowalski and Mulak, 2019
*Bifidobacterium bifidum Bb*	Human normal subjects	Increase in Ruminococcaceae and a decrease in Prevotellaceae, further increasing fecal butyrate levels, a helpful SCFA involved in the reduction of neuroinflammation	Gargari et al., 2016
Fructo-oligosaccharides (FOS)	Male APPswe and PS1ΔE9 mutant transgenic mice	Increase in GLP-1 when compared to AD-mice, promoting satiety and slowing gastric emptying and forestalling CNS insulin resistance, thus decelerating neuronal cell death. Also, FOS lead to an increase in synapsin-1 when compared to the diminished expression of the protein in AD mice, resulting in normal neuronal activity	Sun et al., 2018
Xylo-oligosaccharides (XOS)	APP/PS1 mice	Significantly increased levels of Muribacterium and Lactobacillus in POCD mice, genera that were deficient in POCD-APP/PS Mice. Attenuated levels of the pro-inflammatory cytokines IL-6, of IL-1β, and the immunosuppressive cytokine IL-10. Also increased ZO-1 levels in epithelium and hypothalamus tissue countering the leaky gut and leaky brain phenotype of AD-mice. Restored TREM2 levels, leading to the normalization of microglia neuroinflammatory response. Induces growth o*f Bifidobacterium adolescentis* and *Bifidobacterium longum*, the former being documented for its neuroinflammatory properties	Ávila et al., 2020; Han et al., 2020
Polyphenols (Ferulic Acid)	Female APPswe and PS1ΔE9 mutant transgenic mice	Acts as a ROS scavenger, diminishing neurotoxicity. Inhibits formation of Aβ fibrils at the cortical and hippocampal level. Decrease in IL-1β pro-inflammatory cytokine	Yan et al., 2001
Triphala (TFLA)	–	Reduces neuronal deterioration, improved longevity, motility, reduced Aβ accumulation, and increased acetylcholinesterase activity	Westfall et al., 2019
**Synbiotics**			
*Lactobacillus acidophilus*, *Bifidobacterium bifidum*, and *Bifidobacterium longum* and *selenium*	UAS-(β-secretase) BACE1-APP AD-induced *Drosophila melanogaste*r	Same effects as mentioned above for triphala, albeit more potent in every category, especially Aβ accumulation and acetylcholinesterase activity	Westfall et al., 2019
*Lactobacillus plantarum* NCIMB 8826, *Lactobacillus fermentum* NCIMB 5221 and *Bifidobacteria longum* spp. infantis NCIMB 702255 and Triphala	Human AD patients	Improvement in MMSE score, reduced hsCRP in plasma, and reduced plasma total antioxidant capacity and glutathione. Markers of insulin metabolism and dyslipidemia were also decreased by the supplementation of the synbiotic	Tamtaji et al., 2018
Kefir	Human Ad patients	Notable improvement in all cognitive tests: memory, visual-spatial, executive function and language due to the decreease in ROS compounds such as O_2_^–^, H_2_O_2_, ONOO^–^/OH^–^, and the increase in ROS scavenging compounds such as NO. Also, synbiotic supplementation lead to a decrease in pro-inflammatory cytokine expression, and an increase in anti:pro-inflammatory ratio	Ton et al., 2020
*Lactobacillus paracasei* HII01 and XOS	Male Wistar obbese insulin-resistant rats	improved cerebral function by mitigating gut inflammation, hippocampal oxidative stress and microglial activation	Chunchai et al., 2018

### Effects of Prebiotics on AD

Dietary polyphenols such as FA, as well as different non-digestible fibers such as inulin and oligofructose, are capable of maintaining a healthy gut microbiota by promoting the growth of commensal strains and inhibiting the growth of pathogenic bacteria (Lupien-Meilleur et al., 2016; Constante et al., 2017). These dietary compounds are referred to as prebiotics, and have been shown to confer many benefits that mitigate progression of neurodegenerative disease.

One of the most studied prebiotic compounds is fructooligosaccharide (FOS), found naturally in many fruits and vegetables and enzymatically derived from inulin degradation processes. As a dietary supplement, FOS promotes the production of *Lactobacillus and Bifidobacterium* by serving as a substrate for their proliferation. The effects of FOS on cognitive impairment have been investigated by many groups, whose results in pre-clinical mice studies endorse it as a potential food supplement to combat neurodegeneration. In a study by Sun et al. (2018) FOS supplementation to transgenic AD mice lead to an increase in Glucagon-like peptide-1 (GLP-1), a protein which readily crosses the BBB and promotes satiety, pancreatic secretions of insulin and slowing of gastric emptying. This increase in cerebral GLP-1 forestalls CNS insulin resistance, thus decelerating neuronal cell death from the impaired glucose metabolism seen in AD subjects. FOS supplementation has also been shown to influence neuroplasticity via expression of synapsin-1, a presynaptic protein that coats synaptic vesicles transporting neurotransmitter, and acts as an indicator for neuronal activity. Although synapsin-1 levels experience a considerable decrease in AD subjects, a supplementation of FOS in AD mice lead to restoration of physiologically normal synapsin-1 levels compared to the controls.

**FIGURE 3:**
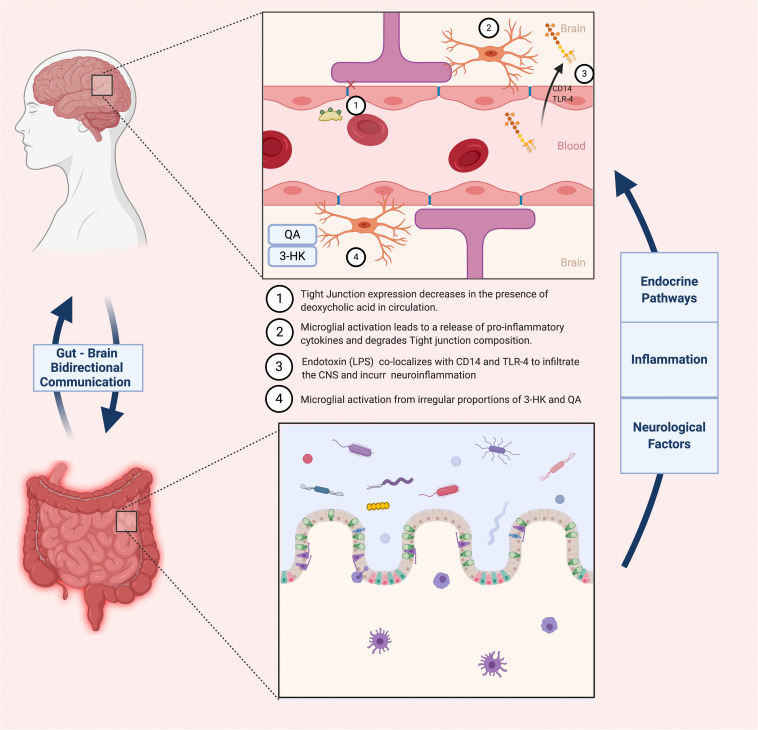
Contrasts the healthy vs. diseased states of the BBB, and how the gut microbiota impacts these states.

A similar oligosaccharide that has been extensively studied and generated equally promising results is xylooligosaccharides (XOS). This prebiotic is sourced from β-1,4 linked units of xylose, forming oligomers of xylan. XOS is the most abundantly present biopolymer in the plant kingdom, and can be naturally derived from fruits, vegetables, bamboo sprouts, honey or most recently, sugar cane biomass (Ávila et al., 2020). Its ease of access, in combination with its documented anti-inflammatory properties, inspired several studies researching its potential effects on cognitively impaired subjects. This notion was tested by the group of Han et al. (2020) by administering XOS supplements to APP/PS1 mice suffering from hepatectomy-induced postoperative cognitive dysfunction (POCD), a common comorbidity of AD. Typical symptoms of POCD include memory loss, lack of balance and executive functions due to neuroinflammation and a reduction in BBB integrity. Post-surgery microbiome analysis showed that three genera were significantly increased (*Rodentibacter, Bacteroides* and *Ruminococcaceae*) and two were decreased (*Faecalibaculum* and *Muribaculaceae*). Increased amounts of *Ruminococcaceae* have been associated to cognitive dysfunction when analyzing fecal samples of AD-induced rats, although this relationship is not entirely consistent across studies. On the other hand, an increase *Faecalibaculum* has been shown to markedly impact fecal SCFA levels in AD patients, limiting systemic and thus neuroinflammation (Biagi et al., 2010; Claesson et al., 2011). Supplementation of the prebiotics to the operated mice attenuated the microbiota fluctuations experienced post-surgery, most significantly in the *Faecalibaculum*, *Bacteroidetes*, *Ruminococcaceae*, *Lactobacillus* and *Muribaculaceae* genera. Furthermore, augmenting the richness of the gut microbiota through XOS administration attenuated intestinal inflammation, decreasing levels of the pro-inflammatory cytokines IL-1β and IL-6 as well as the immunosuppressive cytokines IL-10, all of which were previously increased by the hepatectomy. As previously mentioned, downregulation of tight junctional proteins at the epithelial barrier and BBB is a major marker for the pathology of neuroinflammation. Following POCD, AD mice expressed an extensive weakening of both barrier functions, exhibited by a decrease in tight junction proteins zonulin-1 (ZO-1). These specific junctional proteins bind to occludins at the actin cytoskeletal level so as to horizontally link adjacent cells and strengthen the barrier separating the gut from the systemic circulation (Hassanisaber et al., 2019). Supplementation with XOS led to an upregulation of ZO-1 in both epithelial and hypothalamic tissue, indicating a potential relationship between the “leaky gut” phenotype and a more permeable BBB. To further validate these findings, expression levels of TREM2 proteins (regulators for microglia polarization and CNS inflammation) were quantified, and hippocampal regions of AD mice were inspected for alterations in BBB microstructure using transmission electron microscopy (TEM). Indeed, initial results were corroborated by both experiments. Following a deficiency in TREM2 levels and somatic enlargement of microglia during POCD, supplementation with XOS restored TREM2 concentrations and lead to a reduction of the microglia-mediated neuroinflammatory response. Furthermore, TEM imagery of the BBB provided visual evidence that the supplemented AD mice showed a comparable BBB composition and structure to control subjects. A summary of microbiota-related effects on BBB integrity is summarized in Figure 3.

In contrast to other prebiotics such as FOS, XOS demonstrates a better resistance to digestion, and an increase in gut *Bifidobacteria* diversity (Tungland, 2018). Indeed, not only does consumption of XOS promote the production of *Lactobacilli* and *Bifidobacteria* genera, but it has been shown that certain strains such as *Bifidobacterium adolescentis* and *Bifidobacterium longum* grow more effectively in XOS-supplemented individuals over FOS-supplemented individuals. Importantly, *Bifidobacterium adolescentis* has been documented for its impact on neuroinflammation (Guo et al., 2019). Finally, synbiotic formulations have harnessed the potential of XOS in combination with probiotic microbial strains known to selectively ferment this prebiotic substrate. The group of Chunchai et al. (2018) formulated a synbiotic supplement containing XOS and *Lactobacillus paracasei* HII01, and supplemented obese-insulin-resistant rats with the prebiotic, probiotic or synbiotic in order to note any divergent effects. The rats fed with the prebiotic, probiotic and synbiotic formulations all improved cerebral function by mitigating gut inflammation, reducing oxidative stress in the hippocampus and alleviating microglial activation. The study spawns an interesting conjecture. Although prebiotics generate positive results when supplemented in AD, the coalescence with probiotics may engender superior benefits in mitigating AD markers.

### Probiotics With Prebiotic Formulation Shows Superior Benefits in AD

Synbiotics were first conceptualized in 1995 by Gibson and Roberfroid, 1995 and have since accumulated a plethora of research acknowledging the benefits of a combined pre- and probiotic supplement. Among this research, one notable study was conducted on a transgenic humanized *Drosophila melanogaster* model with a BACE1-APP induced-AD phenotype (Westfall et al., 2019). The insects were either supplemented with three metabolically active probiotics (*Lactobacillus plantarum* NCIMB 8826, *Lactobacillus fermentum* NCIMB 5221 and *Bifidobacterium longum* spp. *infantis* NCIMB702255), the polyphenol-rich plant prebiotic Triphala (TFLA) or the synbiotic that combined strain and substrate. The strains which constituted the probiotic mixture were selected due to their inhibition of Aβ aggregation, decrease in neuroinflammation and their ability to produce a secondary source of antioxidants (Nimgampalle and Yellamma, 2017; Li et al., 2019). *Lactobacillus fermentum* produces FA, whose anti-inflammatory and anti-AD properties have already been well explored in the previous section. *Lactobacillus plantarum* enhances *Lactobacillus fermentum*’s therapeutic properties by metabolizing FA into caffeic and vanillic acid, compounds that further increasing the anti-inflammatory properties of the mixture and add a neuroprotective effect against fibril formation. Finally, the prebiotic TFLA has a considerable concentration of yet another organic acid, gallic acid, which has been shown to reduce neuronal deterioration and promote a favorable gut microbiota (Vidhya Rekha et al., 2019). Results demonstrated the superior benefit of a combinatorial approach over the sum of its parts. Over the duration of the study the synbiotic mixture had a positive effect on longevity, motility, Aβ accumulation and acetylcholinesterase activity, all biomarkers of AD, signifying that the combination might be a potential therapeutic for the disease. Indeed, *Drosophila melanogaster* survivability increased substantially when treated with TFLA or probiotics, but the effect was strongest when both were combined as a synbiotic. An equivalent improvement in motility and Aβ accumulation was seen for the individual and combined treatments. Peculiarly, when compared to the administration of the probiotic mixture or the prebiotic alone the synbiotic effect wasn’t distinctly better on any given test, but maintained a consistently high benefit across tests compared to its counterparts. As is the main premise of the paper, AD does not display a single targetable symptom and biomarker, suggesting that multiple factors could influence its onset. Although probiotics and prebiotics individually demonstrated potential, the consistency of a combinatorial treatment might prove to be a more useful solution for such a multi-faceted disease.

Human clinical trials are a rarity when it comes to synbiotics due to the infrequent comparison of sole supplementation versus combination supplementation within a single study. Although most clinical studies performed for synbiotics on neurodegenerative disorders remain inconclusive, most likely due to the high variety in cohorts, some research has demonstrated promising results. The group of Tamtaji et al. (2018) investigated the metabolic anti-inflammatory and antioxidative profiles of 79 AD patients before and after the administration of a synbiotic formulation. Patients were randomly supplemented with either the probiotic mixture combined with selenium, selenium alone or a starch placebo. The probiotics utilized in this study were *Lactobacillus acidophilus*, *Bifidobacterium bifidum*, and *Bifidobacterium longum*, due to their documented anti-inflammatory properties and antioxidative benefits. Selenium on the other hand, is an important trace element known for its antioxidant properties, and has already shown promise in the prevention of the onset of AD (Hatfield et al., 2016). The study established that the co-supplementation of selenium with the probiotics, when compared to the administration of selenium alone or the placebo, had significant effects on the patient’s cognitive ability, indicated by an improvement in the MMSE score. Additionally, metabolic markers of inflammation and oxidative stress such as CRP, plasma total antioxidant capacity (cumulative effect of plasma antioxidants) and oxidized glutathione were markedly reduced. Markers of insulin metabolism and dyslipidemia such as triglycerides, VLDL-, LDL-, and HDL-cholesterol also experienced a significant reduction following synbiotic administration. Although the cohort supplemented with selenium alone showed favorable results when compared to the control/placebo group, the results were substantially less advantageous than those seen in their synbiotic-supplemented counterparts, supporting the postulation that synbiotics can be equally if not more advantageous than prebiotics or probiotics alone. Similar evidence regarding the benefits of a synbiotic formulation was studied by the group of Ton et al. (2020) in which they investigated the effects of probiotic-fermented kefir milk in terms of oxidative stress, inflammation and cognition. When supplemented with probiotic-fermented kefir, AD patients displayed a notable improvement in all cognitive tests. Similar to a previously mentioned study performed by Akbari et al. (2016) which also examined the benefits of probiotic milk derivatives, all subjects administered kefir supplementation showed marked improvements in memory as well as visual-spatial, executive, and language functions. Furthermore, the present study monitored the differences in plasma intracytoplasmic ROS, such as O2_-_, H_2_O_2_, ONOO^–^/OH^–^, and the amount of free radical scavenging compounds such as NO. These measurements revealed that kefir supplementation in AD patients decreased ROS and increased NO levels in blood. This suggests that the kefir supplement may diminish protein oxidation (and subsequent cell death) due to its potential antioxidative properties. Additionally, supplemented patients showed diminished expression of pro-inflammatory cytokines such as IL-8, IL-12 and TNF-α, and an improvement in the anti-/pro-inflammatory cytokine ratio overall. Although these results solidify the aforementioned effects of synbiotics, the small sample size of 16 patients must be considered as a limitation for the study. All and all, synbiotic formulations provide superior bioavailability of microbially produced anti-inflammatory and antioxidant metabolites, offering a biological tool that may enhance the benefits of microbiome modulation for host physiology.

### Influence of Diet and Lifestyle in AD

In the United-States, Alzheimer’s Disease prevalence saw an increase of 89% between the years of 2000 and 2014 Alzheimer’s Disease (2019). Considering the non-infectious nature of the disease, this substantial rise in cases must be attributed to a significant disruption in environmental factors. A common hypothesis for the underpinnings of this trend is related to the role of day-to-day diet on neurodegeneration and neurodevelopment. The lack of fiber and healthy fat intake could induce a state of systemic chronic inflammation by altering one’s microbiome composition drastically, subsequently affecting the brain through the MGBA (Bengmark, 2013). Recently, scientists from the European Molecular Biology Laboratory (EMBL) and the MetaHIT Consortium categorized various diet-induced gut microbiota into three subcategories, termed “enterotypes” (Costea et al., 2018). The three enterotypes, namely, the *Bacteroides*, *Prevotella* and *Ruminococcus* enterotypes, are predominantly composed of their namesake bacteria genus. These enterotypes were classified based on their ability to ferment and digest the main nutrients in human diets. For example, the *Bacteroides* enterotype occurs on a diet mainly composed of carbohydrates, fibers, fructans, and animal protein, with a serum overexpression of CRP and low-grade inflammation (Smati et al., 2015). The *Prevotella* enterotype is enriched with heavy fiber diet, mainly plant polysaccharides and mucin glycoproteins (De Wouters et al., 2013). Finally, the *Ruminococcus* enterotype (or *Firmicute* enterotype) is established if one’s diet is mainly composed of sugars and animal fats (Robles Alonso and Guarner, 2013). Enterotypes are independent of age, nationality, body mass index and sex, and have become a worldwide recognized tool for characterization of one’s gut microbiota resulting from a consistent dietary pattern. There is no one enterotype considered ideal, since a balanced, diverse gut microbiota remains the strongest consistent marker for overall GI health (Veljovic et al., 2017; Noh et al., 2018). Enterotypes and diet must obey a symbiotic relationship, in which the appropriate diet is harmonized with the coinciding bacterial population so as to produce the optimal quantities of gut-derived metabolites. Indeed, the probiotic *Bifidobacterium bifidum Bb* was utilized as an intervention in a randomized, double-blind, crossover, and placebo-controlled clinical study in which the probiotic was tested for its ability to durably alter the gut microbiota composition of individuals with different enterotypes (Gargari et al., 2016). After 4 weeks, the probiotic-treated group had a transformed microbiota, such that 8 of the subjects changed enterotype during the study. Furthermore, supplementation of *B. bifidum Bb* led to an increase in *Ruminococcaceae* and a decrease in *Prevotellaceae*. This alteration further increased fecal butyrate levels; a metabolite of gut microbes, cells and stimulates gene host expression, immune system and host-brain communication through the vagal nerve (Stilling et al., 2016). Coincidently, fecal analysis of AD elders displayed a microbiome lacking butyrate-synthesizing bacteria, leading to an increased pro-inflammatory state (La Rosa et al., 2018). Overall, these results demonstrate that while diet may be a key factor in establishing enterotype, the microbiome is dynamic and capable of transitioning between steady, stable states with sufficient intervention.

The debate surrounding an ideal diet composition is ongoing and controversial, although to date the results are inconclusive and generalized. The Western diet, characterized by a daily high fat and high sugar intake, has been associated with cognitive deterioration due to its negative impacts on the microbiome and related profiles of bacterial metabolites (Martinez et al., 2017). Indeed, the incidence of AD increased in countries with a higher average caloric intake and adherence to westernized dietary habits (Kalaria et al., 2008). High sugar and high fat diets led to a decrease in short-chain fatty acids (SCFA) metabolites from the gut microbiota. As aforementioned this results in a hyperpermeable gut epithelial membrane and triggers a pro-inflammatory response due to an increased exposure of foodborne toxins in systemic circulation. On the other hand, a staple diet which rectifies SCFA deficiency is the Mediterranean diet: composed of plant-based foods such as fruits, vegetables and whole grains, along with a small amount of seafood and eggs (Nagpal et al., 2019). A cross-sectional study investigated the prevalence of AD in subjects adhering to the Mediterranean diet, and demonstrated that such a diet increased the patient’s MMSE over 18 months independently of their sex, country of origin, education and age (Gardener et al., 2012). Indeed, a proposed mechanism for this cognitive benefit involved a decrease in gut and neural inflammation due to an altered, more equilibrated microbiome. In a similar study conducted by the University of Auckland, a 6-week intervention with a Mediterranean diet equivalent resulted in increased levels of *Bacteroidetes* and *Clostridium* spp. across subjects as well as a decrease in *Proteobacteria* and *Bacillaceae*, effectively leveling the gut microbiota to normal amounts when contrasted to healthy control individuals (Marlow et al., 2013). Understanding which foods promote a balanced microbiome and which nutrients aid in maintaining said microenvironment is at key to enhancing and optimizing microbiota gut-brain communication. Thus, the impact of diet and lifestyle on neurodegeneration via the MGBA must be further researched, in order to mitigate the environmental factors that are so fundamental to the etiopathogenesis of the disease.

## Limitations of Probiotics, Prebiotics and Synbiotics as Therapeutics for Alzheimer’s

Although available data suggests significant therapeutic potential of probiotics in AD, many barriers remain to be overcome before probiotic treatment is endorsed in medical practice. Currently the United States Food and Drug Administration (US FDA) has approved a select list of probiotics, which are known to be considered as safe for commercial use in food and in probiotic supplements (Bhadoria and Mahapatra, 2001). However, the FDA has not approved any claims for probiotics that relate them to a reduction in the risk of disease or as a viable treatment for extant medical conditions (Teshale et al., 2017). Indeed, in addition to the numerous health benefits of probiotics there are risks and uncertainties associated with their use. Numerous reports have connected probiotic use to deleterious effects including sepsis, immunoreactivity, and gene transfer resulting in pathogenic antibiotic resistance (Boyle et al., 2006; Teshale et al., 2017). These risks are of highest concern with respect to vulnerable groups including the elderly, critically ill and immunocompromised, making probiotic use in the context of pathology especially prone to complications. Furthermore, the highly strain-specific effects of probiotic supplementation make it susceptible to pleiotropic or unanticipated metabolic outcomes. As such, there is an egregious lack of consensus in the literature and the field regarding the appropriate formulation, dose and treatment schedule that will maximize patient outcome while minimizing collateral effects.

Another limitation of probiotic supplementation in AD involves their transient, non-predictable colonization of the gut mucosa. Indeed, depending on the individual, some probiotics may establish poorly into an already stable gut microenvironment. Particularly, humans have a selectivity mechanism for probiotics wherein gut mucosa resistance to probiotics is specific to the individual. This makes it difficult to pinpoint which probiotic of a given multi-probiotic supplementation impacts the given results (Zmora et al., 2018). Furthermore, at times, the overproduction of serotonin (5-HT) by the gut microbiota can give rise to the serotonin syndrome in subjects. Serotonin syndrome is often a result of selective serotonin reuptake inhibitor medicine in depression, where an excess in serotonin agonism leads to a surplus in 5-HT receptors in the CNS and in the periphery (Feeney, 2007). Alternatively, sole supplementation of tryptophan-metabolizing probiotics can, but rarely result in serotonin syndrome, but its combination with potent anti-depressants may lead to hind limb abduction, head weaving or other symptoms of the serotonin syndrome.

Overall, in order to be regulated at the level of a pharmaceutical or biological product, further work remains to be done to ensure probiotic interventions meet standards of safety, purity, and potency appropriate for medical applications. By further understanding the mechanisms underlying both the benefits and detriments of probiotics in host health, there is opportunity to generate safe, targeted treatment methods that maximize their potential in combatting AD.

## Perspectives and Conclusion

Twenty-first century disorders such as anxiety, depression, obesity, diabetes, Parkinson’s disease, and AD appear completely disparate in nature, but in fact share two key commonalities. Firstly, they are all non-transmissible, non-communicable diseases or disorders that currently lack the appropriate medication to fully eradicate their symptoms. Secondly, there exists extensive literature linking these disorders to perturbations of the microbiome. Several probiotics, prebiotics and synbiotics have been shown to enhance microbiome stability, with subsequent benefits to brain health that are particularly useful in combatting neurodegenerative pathology such as AD. Although AD’s etiology remains poorly understood, probiotics, prebiotics and synbiotic supplementations have shown the ability to decelerate or potentially reverse the disease’s main symptoms through several mechanistic avenues. Whether by alleviating the state of chronic neuroinflammation, scavenging peripheral and cerebral ROS, altering the production and localization of neurotoxic metabolites, or adjusting energy metabolism to serve the vital demands of neurons, modulating the microbiome through these bioactive supplements has shown promising results. Considering how much literature has emerged supporting probiotics, prebiotics and synbiotics as potential solutions to AD, there is much work to be done in elucidating their mechanisms of action and further exploring the overlap between AD and microbiome research.

The microbiome and its probiotic bacteria have played an intrinsic role in human health throughout evolution, and not taking care of it or ignoring its therapeutic potential would be to neglect our genetic majority (Dietert, 2016). Extensive relationships between these bacteria and our digestive, immune and nervous systems is far from delusion- and these principles must be applied to our current view of neurodegenerative disease to develop safe, effective, and targeted probiotic therapies.

## Author Contributions

KA and SP: conceptualization. KA: data curation and visualization. SP: funding acquisition, project administration, resources, and validation. KA, MG, and SP: methodology and writing, reviewing, and editing. KA and MG: software and writing – original draft. All authors contributed to the article and approved the submitted version.

## Conflict of Interest

KA was employed by the company Biena Inc. The remaining authors declare that the research was conducted in the absence of any commercial or financial relationships that could be construed as a potential conflict of interest.
